# Genome Sequence of EU-Unauthorized Genetically Modified *Bacillus subtilis* Strain 2014-3557 Overproducing Riboflavin, Isolated from a Vitamin B2 80% Feed Additive

**DOI:** 10.1128/genomeA.00214-15

**Published:** 2015-04-09

**Authors:** Elodie Barbau-Piednoir, Sigrid C. J. De Keersmaecker, Véronique Wuyts, Céline Gau, Walter Pirovano, Adalberto Costessi, Patrick Philipp, Nancy H. Roosens

**Affiliations:** a Platform Biotechnology and Molecular Biology, Scientific Institute of Public Health (WIV-ISP), Brussels, Belgium; b Service Commun des Laboratoires, Illkirch-Graffenstaden, France; c Baseclear B.V., Leiden, the Netherlands; d Department of Microbial and Molecular Systems, KU Leuven, Leuven, Belgium

## Abstract

This paper announces the genome sequence and annotation of the genetically modified (GM) *Bacillus subtilis* strain 2014-3557 overproducing riboflavin (vitamin B2). This GM-strain is unauthorized in the European Union. Nevertheless, it has been isolated from a lot of vitamin B2 (riboflavin) 80% feed grade imported to Europe from China.

## GENOME ANNOUNCEMENT

Riboflavin (vitamin B2) cannot be biosynthesized by vertebrates, whereas plant and most microorganisms are able to do so (1). Therefore, vitamin B2 is used as a food and feed additive. As an alternative to costly chemical synthesis, microbial fermentation processes of riboflavin were developed for industrial production. Naturally producing or riboflavin-overproducing microorganisms transformed by a genetic engineering, chemical, or physical process can be used. *Bacillus subtilis*, a Gram-positive, rod-shaped bacterium, is exploited in industry for this purpose (1–3).

In July 2014, Germany detected a viable *Bacillus subtilis* strain which harbored a non-naturally occurring combination of DNA sequences in a lot of vitamin B2 feed additive imported from China. This strain was considered as genetically modified and unknown; therefore it is unauthorized in the European Union. In September 2014, the European Rapid Alert System for Food and Feed (RASFF) created a notification to alert the other European countries about the presence of the unauthorized GM-*Bacillus subtilis* in this particular vitamin B2 feed additive (https://webgate.ec.europa.eu/rasff-window/portal/ [enter reference 2014.1249]).

Consequently, the French competent authorities investigated this kind of product imported in France. A French National Reference Laboratory (NRL) for GMO isolated a yellow substance (presumably overproduction of riboflavin)-secreting bacterial strain from three samples of vitamin B2 feed additives imported from China.

To further characterize this finding, whole-genome sequencing was performed on one of the three isolates with an Illumina HiSeq2500 run using a paired-end library. Sequencing yielded 10,914,314 paired-end reads (350-fold coverage), which were assembled *de novo* using CLC Genomics Workbench version 7.5.1 (CLC Bio). The resulting draft genome was further linked into scaffolds with SSPACE (4) based on paired-end read linkage. Finally, a total of 39 gap-closed scaffolds were generated consisting of 143 contigs with a maximum gap-closed scaffold size of 1,018,461 bp and a minimum size of 370 bp. After filtering of these 39 scaffolds, three were discarded as two matched with *Homo sapiens* and one with *Haemonchus placei*. The total sequence length post-filtering is 4,175,764 bp, and has a G+C content of 44.32%.

Genome annotation was performed on the assembled scaffold sequences using the BaseClear annotation pipeline which is based on the Prokka Prokaryotic Genome Annotation System. This annotation confirmed the organism as *Bacillus subtilis* with 100% identity. 4,233 genes were predicted, of which 4,144 have a known function, including several encoding for proteins involved in riboflavin synthesis and transport. The genome sequence of this *Bacillus subtilis* strain will be useful for further characterization of its GM nature and in developing a specific method for its detection in food and feed additives by enforcement laboratories.

### **Nucleotide sequence accession number**s. 

This whole-genome shotgun project has been deposited at DDBJ/EMBL/GenBank under the accession no. JYFL00000000. The version described in this paper is version JYFL01000000.
